# Ultrasonography for the assessment of lung recruitment maneuvers

**DOI:** 10.1186/s13089-016-0045-9

**Published:** 2016-08-05

**Authors:** Gerardo Tusman, Cecilia M. Acosta, Mauro Costantini

**Affiliations:** Department of Anesthesiology, Hospital Privado de Comunidad, Córdoba 4545, 7600, Mar del Plata, Buenos Aires Argentina

**Keywords:** Lung ultrasound, Recruitment maneuvers, Tidal recruitment, Atelectasis, VILI

## Abstract

Lung collapse is a known complication that affects most of the patients undergoing positive pressure mechanical ventilation. Such atelectasis and airways closure lead to gas exchange and lung mechanics impairment and has the potential to develop an inflammatory response in the lungs. These negative effects of lung collapse can be reverted by a lung recruitment maneuver (RM) i.e. a ventilatory strategy that resolves lung collapse by a brief and controlled increment in airway pressures. However, an unsolved question is how to assess such RM at the bedside. The aim of this paper is to describe the usefulness of lung sonography (LUS) to conduct and personalize RM in a real-time way at the bedside. LUS has favorable features to assess lung recruitment due to its high specificity and sensitivity to detect lung collapse together with its non-invasiveness, availability and simple use.

**Electronic supplementary material:**

The online version of this article (doi:10.1186/s13089-016-0045-9) contains supplementary material, which is available to authorized users.

Positive pressure mechanical ventilation is a non-physiological intervention that saves lives but is not free of important side effects. It invariably results in different degrees of collapse of both, small airways and/or the whole acinus. The resulting loss of lung aeration can range from local hypoventilation to complete atelectasis [1, 2]. The consequences include not only gas exchange deterioration and lung mechanics impairment but also an enhanced risk of triggering an inflammatory response in the lungs [3–5]. Importantly, this local inflammation-termed ventilator-induced lung injury (VILI)—has been described to occur in both, ARDS and healthy patients [5–7].

Lung protective ventilation is aimed at decreasing VILI by limiting tidal volume and driving pressure [plateau pressure-end-expiratory positive pressure (PEEP)] [6, 8, 9]. Such an approach minimizes the impact of the two known VILI mediators: tidal over distension (i.e. alveoli that receive volume and pressure that exceed their elastic limit) and tidal recruitment (i.e. the repetitive opening and closing of atelectasis during mechanical breathing) [10–13].

The Open lung approach is another ventilatory strategy complementary with the concept of protective ventilation [14–16]. Lachmann was the first who introduced the open lung concept combining a lung recruitment maneuver (RM) with a sufficient level of PEEP [16]. RM aims to resolve lung collapse by a brief and controlled increment in airway pressure while PEEP afterward keeps the lungs open. It not only improves lung mechanics and gas exchange but also can minimize VILI as tidal recruitment cannot occur if atelectasis is eliminated and tidal over distension can be minimized in a more homogenous lung combined with a low driving pressure [12–15].

Physiology determines that each alveolus has a closing and an opening pressure threshold [14, 16, 17]. As gravity results in a ventral-to-dorsal gradient of trans-pulmonary pressures along the lungs, collapse will predominate in the most dependent (dorsal) regions where trans-pulmonary pressure are lowest at end expiration.

The success of such a ventilatory strategy critically depends on the right selection of opening and closing pressures. Otherwise patients can be submitted to undesirable high or inadequate low airways pressures. Therefore, ideally RM and PEEP selection must be individualized and this can only be done when guided by specific monitoring tools since lung’s opening and closing pressures vary among patients with different lung conditions [14, 17].

Taking this into account, ultrasound has favorable features to assess RM due to its high specificity and sensitivity to detect lung collapse together with its non-invasiveness, availability and simple use at the bedside [18]. Figure 1 illustrates how ultrasound can guide a clinical RM based on a four step algorithm: two related to the “assessment” before the maneuver and two to the “confirmation” of the results of lung recruitment. The first question we must answer is whether our patient needs a RM—step 1. This question is important because impairment in pulmonary function may have different causes other than lung collapse. Thus, a complete lung ultrasound scan based on the standard three-region examination in each hemithorax must be done to diagnose the patient’s lung condition [18]. Ultrasound can detect the presence of lung collapse predominantly in dependent (dorsal) pulmonary areas following the gravity vector. Lung collapse can manifest as slight to moderated loss of lung aeration (from isolated to coalescent B-lines) up to complete atelectasis (sub-pleural consolidations with static air-bronchograms) [18, 19]. Such areas can be quantified using the aeration score described by Bouhemad et al. [20]. The presence of coalescent B-lines and consolidations together a high aeration score is a clear indication for a RM.

**Fig. 1:**
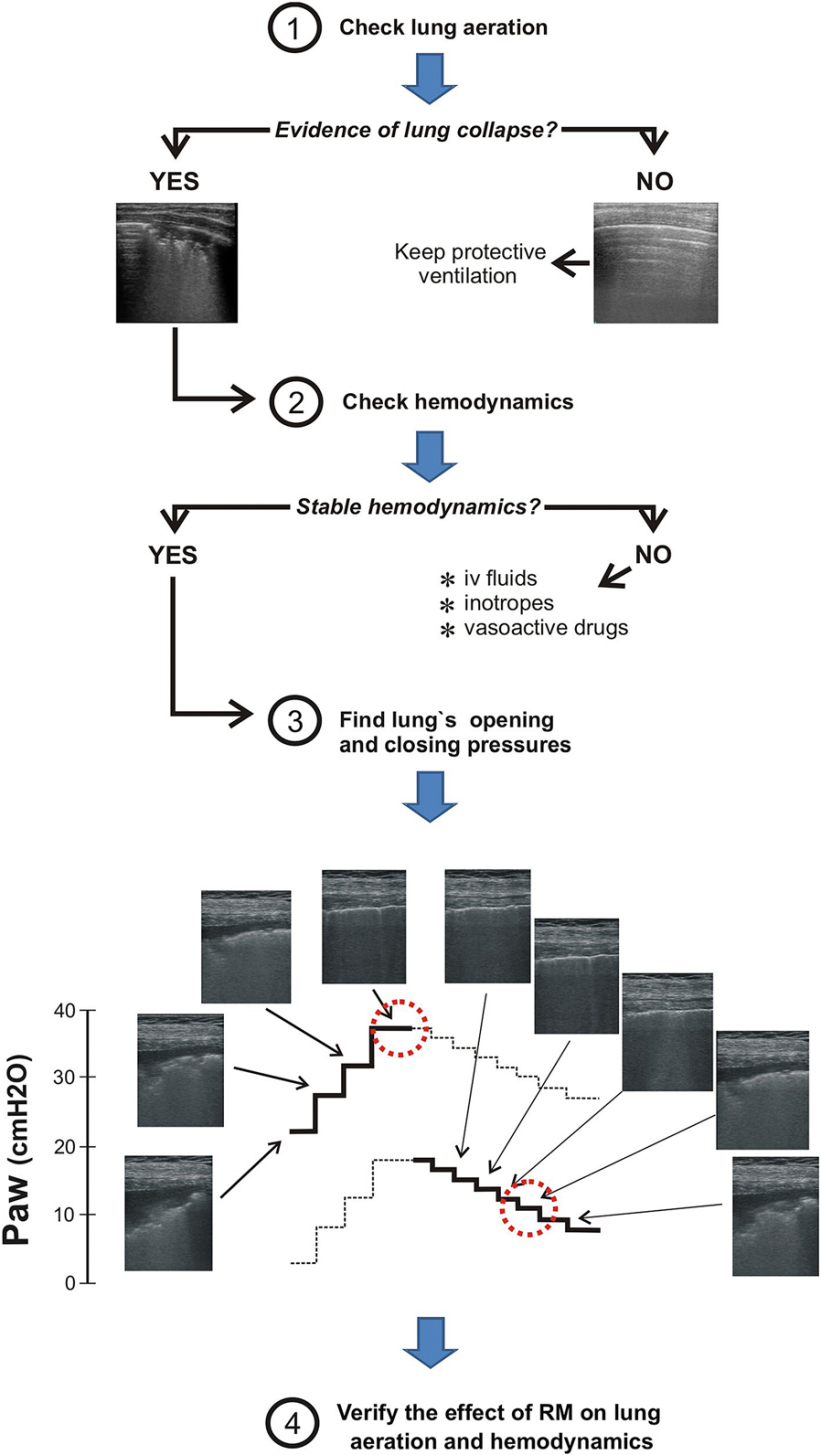
Algorithm to assess recruitment maneuver by ultrasound. Ultrasound diagnoses lung collapse (step 1) and assesses hemodynamics (step 2) before the lung recruitment maneuver is done (step 3). During the step-wise increment in airways pressure (Paw) of the recruitment maneuver, ultrasound can detect the plateau pressure at which dependent lungs collapse disappear (opening pressure—*upper circle*). Then, ultrasound defines the start of lung re-collapse in the most dependent pulmonary areas during the PEEP trial (closing pressure—*lower circle*). The images were obtained in an anesthetized child using a linear 7–13 MHz probe placed in the oblique position of the most dependent lung zones

Ultrasound also has the capability of providing a differential diagnosis between atelectasis and lung consolidation of other origin such as pneumonia. The bilateral distribution of consolidations, presence of static air-bronchograms, images of tidal recruitment within consolidation [21] and absence of a companion pleural effusion strongly support the diagnosis of atelectasis. Furthermore, retrospectively the disappearance of the lung consolidation pattern after a RM confirms the diagnosis.

Once lung collapse is confirmed, step 2 aims to determine if the patient is in clinical condition to tolerate a RM [14, 17]. Patient should be sedated, well adapted to the ventilator and hemodynamically stable. The negative hemodynamic repercussion of the brief RM-associated high intra-thoracic pressure is an important concern about this strategy, especially in lung healthy patients. Thus, the maneuver is contraindicated in patients with preload-dependency, hypovolemia, vasoplegia and with impaired myocardial contractility.

Ultrasound can be of great help to diagnose the hemodynamic status using relatively simple measurements that do not require a specialized training, [17]. The inferior vena cava collapsibility index calculated from the subcostal or transthoracic view can estimate patient’s volemia in an easy way [22]. The parasternal short-axis approach also provides a diagnosis of hypovolemia, by the papillary muscle kiss sign, but in addition can also evaluate left ventricle contractility and ventricular interdependence. The apical and subcostal windows give a global view of right-left cardiac chambers size and myocardial contractility [23]. For the trained physician, advanced ultrasound measurements like left ventricle diastolic function, the respiratory variation in peak aortic velocity or the velocity–time integral of aortic flow in response to fluid can provide more precise information about the hemodynamic and volemic status.

Once atelectasis have been diagnosed and an adequate cardiovascular status confirmed, the patient is ready for receiving a RM—step 3. Ultrasound allows detecting the lung’s opening pressure during the pressure increase and the lung’s closing pressure during the descending limb the PEEP titration trial (Fig. 1 and Additional file 1). In this way, higher than necessary inspiratory pressures to open up the lung are avoided and the lowest level of PEEP necessary to maintain the lungs free of collapse can be identified. In other words, ultrasound allows personalizing the implementation of an open lung strategy increasing its reliability and safety [17].

This step 3 is accomplished as follows: First, standard lung ultrasound scan can be performed using different probes whose selection will depend on patient’s age and body constitution as well as the clinical scenario. Thus, a 3–5 MHz convex probe is commonly used in adults while the best option in children is a 7–13 MHz linear probe due to the high image’s resolution. After performing a standard lung ultrasound scan, the selected probe is placed in the most dependent zone of the atelectatic lung. The RM maneuver is started searching the pressure level at which the consolidation-pattern disappears and progressive lung re-aeration is observed. Re-aeration usually follows a sequential pattern: condensation—B2 lines—B1 lines to normal lung images but, sometimes, the pattern of condensation is transformed directly in a normal pattern [20]. Then, the probe is placed in the same position in the contralateral lung and if a normal aeration is confirmed, this level of airway pressure is defined as the lung’s opening pressure.

After lung recruitment it is necessary to perform a step-wise decrement in PEEP (PEEP trial) to detect the lung’s closing pressure. During this phase the echo probe is kept in the same position (dependent lung) as before. The very moment when normal lung aeration images transform into a B1–B2 pattern and consolidation pattern will define the closing pressure. The final selected level of PEEP is chosen to be 2 cm H_2_O above the detected closing pressure [14, 16, 17]. Once the patient’s opening and closing pressures are known, a new RM is performed reaching these target pressures and the identified PEEP level is set for ongoing ventilation.

The last step 4 is aimed at confirming the effect of RM on lung aeration and on hemodynamics. After applying lung protective ventilation settings the clinician will adjust the hemodynamic treatment to maintain this improved cardiopulmonary condition.

In summary, ultrasound is very useful for monitoring the dynamic nature of the lung recruitment-collapse process in real-time and non-invasively. It helps diagnosing lung collapse, assessing hemodynamics and detecting the actual opening and closing pressures. Ultrasound can thus guide and personalize the implementation of an open lung strategy making it easier, shorter and safer.

## Additional file

10.1186/s13089-016-0045-9 Lung recruitment assessment by ultrasound.
